# Dementia and Mild Cognitive Impairment Identification in Illiterate and Low-Educated People: Systematic Review About the Use of Brief Cognitive Screening Tools

**DOI:** 10.3390/bs15020207

**Published:** 2025-02-13

**Authors:** Jonathan Adrián Zegarra-Valdivia, Brenda Chino, Kuripacha Tituana, Lina Zapata-Restrepo, María Martha Unaucho, Milton Lopez-Norori, Carmen Paredes-Manrique, Nilton Custodio

**Affiliations:** 1 Faculty of Health Sciences, Universidad Señor de Sipán, Km5 Road to Pimentel, Chiclayo 14001, Peru; 2 Center of Cognitive and Computational Neuroscience, Universidad Complutense de Madrid, 28040 Madrid, Spain; brenda.chino@achucarro.org; 3 Achucarro Basque Center for Neuroscience, 48940 Leioa, Spain; 4 Global Brain Health Institute (GBHI), University of California San Francisco, San Francisco, CA 90025, USA; kuri.tituana@gbhi.org (K.T.); maria.unaucho.pilalumbo@gbhi.org (M.M.U.); milton.lopez@gbhi.org (M.L.-N.); 5 Fundación Valle del Lili, Cali 760031, Colombia; 6 Facultad de Ciencias de la Salud, Universidad Icesi, Cali 760031, Colombia; 7 Fundación Alzheimer de Nicaragua, Managua 14124, Nicaragua; 8 Facultad de Psicologia, Universidad Tecnológica del Perú, Lima 15073, Peru; c27709@utp.edu.pe; 9 Instituto Peruano de Neurociencias, Lima 15046, Peru; ncustodio@ipn.pe; 10 Escuela Profesional de Medicina Humana, Universidad Privada San Juan Bautista, Lima 15067, Peru

**Keywords:** cognitive screening test, illiteracy, low education, older adults, dementia, middle cognitive impairment

## Abstract

The rising prevalence of dementia, particularly in low-income and developing countries, highlights the urgent need for effective cognitive screening tools. However, the existing tools often fail to address the unique needs of low-educated and illiterate populations, leading to diagnostic disparities. This review aimed to evaluate cognitive screening tests and domains employed globally to detect mild cognitive impairment (MCI) and dementia in low-educated and illiterate older adults. Following the PRISMA guidelines, Searches were performed in Web of Science, Scopus, and PubMed, targeting studies from January 2000 to 2023 involving adults over 45 years old. Of 1611 studies identified, 27 met the inclusion criteria and underwent pair review. The results revealed that most studies preferred adapting the existing tools to local languages over developing culturally tailored instruments. Twelve cognitive tests specifically designed for low-educated populations were identified, with the Mini-Mental State Examination (MMSE) and Montreal Cognitive Assessment (MoCA) being the most utilized, despite their educational biases. Adjusting the cutoff points improved detection (e.g., MoCA: sensitivity 82.5%, specificity 82%). Notably, the Rowland Universal Dementia Assessment Scale (RUDAS) demonstrated superior performance for low-educated groups (sensitivity 89% and specificity 93%). The findings underscore the critical need for region-specific cognitive batteries that integrate functional assessments, ensuring equitable and accurate diagnosis across diverse educational backgrounds.

## 1. Introduction

Cognitive impairment and dementia are global public health problems affecting an increasingly aging population. The largest group of cases is concentrated in low- and middle-income countries (World Health Organization, 2023), representing a challenge for worldwide health systems. The prevalence of MCI is 12–18% among adults over 65 years of age, and some studies point out an increase in prevalence (Bai et al., 2022; Zegarra-Valdivia et al., 2023a). On the other hand, the annual progression rates from MCI to AD are 10–15%) (Ding et al., 2015; Petersen et al., 2018). In addition, the low educational level has been related as a risk factor for developing cognitive decline (Chino et al., 2022; Oliveira et al., 2019). In 2016, the illiterate global rate was 14% (140 million illiterate) in adults over 65 years old, and Latin America and Caribbean (LAC) countries have the fourth-highest illiteracy rate in the world (UNESCO, 2017).

In this context, successful adaptation to the environment as we age depends on abilities such as memory, attention, executive function, reasoning, learning, and understanding of complex ideas (Matzel & Sauce, 2017). These processes reflect a greater cognitive reserve in old age and have been linked to individuals with higher education, higher intelligence quotient (IQ), and higher professional achievement (Medaglia et al., 2017; Stern, 2017) so high-educated individuals with cognitive decline may perform well in every tasks masking their decline (false negatives), while healthy individuals with low educational background may perform poorly and be mistakenly identified as cases of suspected cognitive decline (false positives), implying that a considerable proportion of vulnerable would be excluded from most of the instruments currently available (Pellicer-Espinosa & Díaz-Orueta, 2022) particularly in LAC countries (Lopera et al., 2023).

An accurate diagnosis of dementia requires a reliable neuropsychological assessment (Parra, 2014). Cognitive assessment tools are commonly used for screening impairment, differential diagnosis, determining disease severity, and monitoring disease progression in patients (Frota et al., 2016). Valid tests are needed for assessing individuals across a wide range of cultures and education (Ardila et al., 1989). This is mainly a challenge in LAC countries, where the most challenging socioeconomic inequality prevails worldwide (Moguilner et al., 2024) and there is evidence that fewer years of education attainment were associated with reduced gray matter volume and lower functional connectivity of key brain areas (Gonzalez-Gomez et al., 2024). In this region, education systems reflect the highly unequal societies in which they are located (UNESCO, 2020). Studies showed that the performance of non-demented subjects (of a wide age range and compared by educational level) was directly affected by literacy in tasks such as naming, comprehension, verbal abstraction, orientation, figure matching, and recognition, and that schooling represented a stronger variable than age (Manly et al., 1999; Ostrosky-Solis et al., 1998) and that valid tests are needed for assessing individuals across a wide range of cultures and education (Ardila et al., 1989).

Most currently used neuropsychological tests in LAC were developed without considering illiteracy (Shim et al., 2015). MMSE and MoCA are the most popular cognitive assessment tools worldwide. Still, they have been shown to have a significant bias related to educational level (Bustamante-Paytan et al., 2024; Scazufca et al., 2009; Yancar Demir & Özcan, 2015), non-culturally developed cognitive tests could be challenging in some populations (i.e., indigenous participants). Thus, culture-free items must be produced. Hence, inequity in reaching out to neurocognitive diagnosis has not been completely solved (Díaz-Orueta et al., 2018).

Considering the broad increase in dementia around the world, the high rates of illiteracy, and the lack of comprehensive cognitive tools sensitive to the educational level (Parra, 2014), the present study aimed to critically review the literature on cognitive assessment tools for screening MCI and dementia in low-educated and illiterate populations focusing in LAC countries.

## 2. Methods

### 2.1. Search Strategy

A comprehensive search strategy was used to identify relevant studies on cognitive assessment related to illiterate and low-educated older adults. We systematically searched multiple electronic databases, including PubMed, Scopus, and Web of Science, using keywords and subject headings related to brief cognitive assessment and illiterate and low-educated older adults. The search terms included “cognitive decline”, “screening test”, “low literacy”, and “validity”; the search was conducted between 1 January 2000, and 3 December 2024. The keyword combinations included in the literature search are shown in Table 1.

### 2.2. Inclusion and Exclusion Criteria

The review included the articles published between 1 January 2000, and 3 December 2024. The criteria used to include the articles were (a) studies with adults over 45 years old; (b) with low education levels (less than four years of formal education), or illiterate; (c) evaluated by the mother tongue or idiom predominant, were included in the review; (d) the evaluation process to group definition has a uniform protocol, carried out in memory clinics or research centers operating in the country where the study is proposed; (e) brief screening test does not last more than 15 min for administration; and (f) the articles indicated diagnostic measurement indicators (content validity, criterion validity, internal consistency, or diagnostic accuracy) and were compared with a gold standard of diagnosis (including the last three versions of the criteria of the Diagnostic and Statistical Manual of Mental Disorders-DSM (DSM-4, DSM-4-TR, and DSM-5); NIA-AA (McKhann et al., 2011); or diagnosis made by a specialist in neurology, psychiatry, and geriatrics or based on an extensive and detailed neuropsychological evaluation).

The exclusion criteria considered in the review were (a) excluding the studies with detailed neuropsychological batteries and (b) brief cognitive tests applied for certain types of cognitive impairment secondary to depression, head trauma, or cerebrovascular, psychiatric, neurological, or metabolic disorders will be excluded.

### 2.3. Screening Process

**Figure 1:**
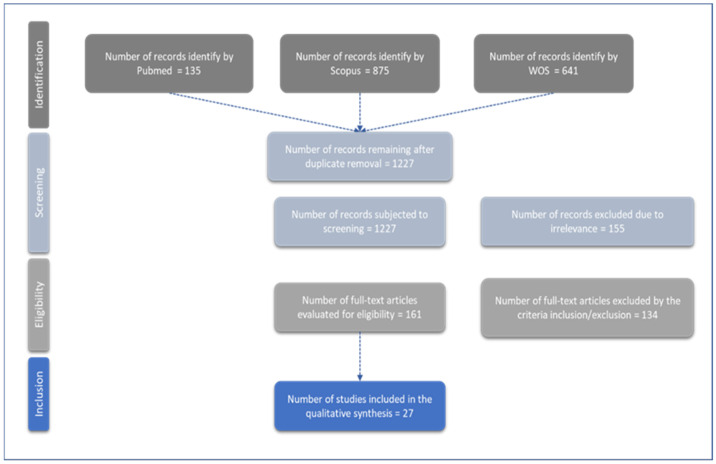
Selection process overview. Flow chart of the included and excluded articles through the screening process following the PRISMA presentation guidelines for the systematic review and for the meta-analysis.

The selected articles were imported into Rayyan (Ouzzani et al., 2016) following the revision process by two reviewers (BC and JZ), as recommended in the Preferred Reporting Items for Systematic Reviews and Meta-Analyses (PRISMA) guidelines for systematic reviews (Shamseer et al., 2015) attending to the selection criteria. The manuscripts selected by the title and abstract review were thoroughly reviewed. Disagreements regarding the selection were resolved through discussion or a third reviewer (screening protocols and final selection are depicted in Figure 1). The third reviewer arbitrated disagreements that could not be resolved by a discussion.

### 2.4. Quality Assessment

The included studies’ quality and risk of bias were assessed using the National Heart, Lung, and Blood Institute (NHLBI) quality assessment tool for observational cohort and cross-sectional studies. Two different reviewers (CP and JZ) applied the abovementioned protocol independently. Discrepancies were solved through discussion or by a third reviewer (BC).

### 2.5. Data Extraction

Data were extracted using a standardized form to ensure consistency and accuracy. The information extracted includes information about the cognitive assessment tool, time duration, country where the study takes place, setting, the dementia criteria used to diagnose participants, sample size, mean age, and female percentage. In addition, educational years and description of participants’ literacy are reported as the diagnostic accuracy (specificity, sensibility, area under the curve for detection purposes, and cutoff points) used in illiterate or low-educated populations of every BCS. Two reviewers (BC and JZ) condensed the data, and a third reviewer checked the selection. The information is summarized in Table S1 (Supplementary file). The BCSs were categorized into cognitive domains and included descriptions of the items to compare and define the differences between the instruments (See Supplementary Tables S2 and S3).

## 3. Results

**Figure 2:**
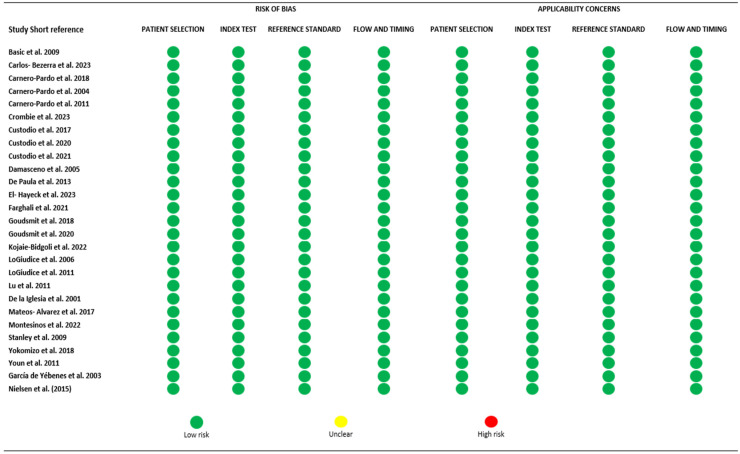
Quality assessment of individual studies using QUADAS tool (Bezerra et al., 2024; Carnero-Pardo et al., 2022; El-Hayeck et al., 2023; Goudsmit et al., 2021; kojaie-Bidgoli et al., 2020; Logiudice et al., 2011; Mateos-Álvarez et al., 2017; Nielsen et al., 2016; de la Iglesiaa et al., 2001).

This study aimed to critically review the literature on cognitive assessment tools for screening MCI and dementia in illiterate and low-educated populations. After conducting a literature search in the specified datasets, 1661 articles were imported for screening. The final studies involved in the review were 27 studies assessed for eligibility to ensure the consistency and accuracy of the articles, considering 17,775 participants for the analysis (see Figure 2).

### 3.1. Brief Cognitive Test Useful for Low-Educated and Illiterate Populations

Fourteenth studies included at least 50% of people with low educational levels, and seven studies (Custodio et al., 2017, 2020b, 2021; Damasceno et al., 2005; de Paula et al., 2013; Goudsmit et al., 2018; Lu et al., 2011) had 100% of participants with ~6 years of education.

Meanwhile, seven studies (Carnero-Pardo et al., 2012; Custodio et al., 2021, 2020b; Damasceno et al., 2005; Goudsmit et al., 2018; Nielsen et al., 2016; Youn et al., 2011) had at least 50% of people with illiterate levels; in three studies (Custodio et al., 2021, 2020b; Goudsmit et al., 2018), 100% of the participants had no level of education, and only one study (Basic et al., 2009) had less than 4% illiterate participants. However, it included 25% who had less than ~6 years of education (in the dementia group, it reached 61%). Regarding psychometric properties, all the studies report sensitivity and specificity, AUC, and cutoff points, except the study about the 37-item version of the MMSE to normative data in a population-based cohort of older Spanish Adults (Contador et al., 2016). After review, seventeen brief cognitive tests were selected. The characteristics and advantages of each test analyzed are described here, such as the domain it measures, whether the test was correlated with other functions or sources of information, and recommended cutoff points.

- The Cognitive Abilities Screening Instrument-Short Form (CASI-S) evaluates attention, concentration, temporal orientation, short-term memory, long-term memory, verbal fluency, visual construction, abstraction, and judgment. The cutoff point for cognitive impairment for adults over 70 years is 20. It has no educational influence; however, in the retrieval subtest, there was a significant difference between illiterates compared to the control group in the Brazilian population (Damasceno et al., 2005).
- Fototest evaluates memory (free recall and facilitated recall), executive capacity (verbal fluency), and naming (language), with the advantage of not being influenced by educational level, so it is applicable in illiterate or low-educational level populations. It is a brief test that can be used in general consultation because it requires 3 min or less for its application (Basic et al., 2009; Carnero-Pardo et al., 2012; Carnero-Pardo & Montoro-Ríos, 2004; Contador et al., 2016; Nielsen et al., 2016).
- The Brazilian version of the General Practitioner Assessment of Cognition (GPCOG-Br) includes a brief cognitive evaluation of the patient and an informant interview. It evaluates temporal orientation, visuospatial skills, episodic memory, delayed recall, and functionality. We analyze the version developed in Brazil in a population with a low educational level, where 25.8% of the participants had no formal education, and 45.16% of the participants had schooling for 1 to 4 years (Yokomizo et al., 2018). The recommended cutoff was a 6/7 total score.
- Informant Questionnaire on Cognitive Decline in the Elderly (IQCODE) is a brief cognitive impairment screening assessment based on an interview with the informant conducted in an Arab population. It assesses memory, the executive capacity of judgment, and functionality. It consists of 16 questions; each is scored on a 5-point scale, and the total sum is averaged over the 16 items assessed. The higher the score, the greater the impairment. The cutoff point for cognitive impairment was >3.34 (Nielsen et al., 2016).
- Kimberley Indigenous Cognitive Assessment-cognitive assessment section (KICA-cog): This short version was developed in Australia for indigenous older adults without schooling. They evaluate cognitive domains such as memory, verbal comprehension, visual nomenclature, recall, and praxis. Its cutoff point is 31/32, with high specificity and sensitivity. It is essential to differentiate it from the original KICA test, which takes 30 to 40 min to assess cognitive functions, functionality, and clinical status by interviewing an informant (LoGiudice et al., 2006).
- Prueba Cognitiva de Leganés (PCL), a 32-item test designed to assess cognitive function in older people with a low educational level, does not require the ability to read, write, calculate, draw, or abstract thinking. The main domains it evaluates are orientation and memory. The cutoff point for the diagnosis of dementia is <22, with high sensitivity and specificity in the Spanish population (De Yébenes et al., 2003).
- Memory Alteration Test (M @ T) is an oral test that does not require reading or writing skills. Its maximum score is 50 points, but its results could be influenced by schooling, which requires adjustment in its cutoff points. It mainly detects memory alterations and can identify healthy people, people with mild amnesic cognitive impairment, and early Alzheimer’s. M@T assesses memory (episodic, textual, and semantic), temporal orientation, free recall, and facilitated recall. The version validated in Peru was carried out in subjects with less than four years of formal education and required adjustment of its cutoff points. Custodio et al. propose cutoff points of 35 and 26 to detect MCI and early AD, respectively (Custodio et al., 2017).
- Mini-Mental Test Examination (MMSE) is one of the most widely used, complete, and disseminated scales globally. Youn et al. tested its applicability in older women with a low educational level, divided into four groups: healthy illiterate, illiterate with mild AD, healthy literate, and literate with mild AD. This classification was performed using the Moon & Chey 2004 illiteracy questionnaire. MMSE originally had a maximum score of 30 points but has shown a high false-positive rate. Youn et al. propose cutoff points adjusted to the education of 24/25 with sensitivity and specificity greater than 90% in the different groups mentioned (Youn et al., 2011).
- Montreal Cognitive Assessment (MoCA) was developed initially to detect MCI and early dementia with high sensitivity and specificity for highly educated people. It is a full brief battery that explores six domains: memory, visuospatial capacity, executive function, language, orientation, and attention/concentration, with a total score of 30 points. Several studies have been developed to demonstrate its efficacy, with positive results towards MoCA; however, for the population with a low educational level, MoCA requires adjustment in its cutoff points. Lu et al. (2011) determined in the Chinese population that the most appropriate MoCA cutoff point for people without formal education was 13/14. The most appropriate MoCA limit for people with 1 to 6 years of education was 19/20 (18). MoCA is the most widely used brief cognitive test in LA, especially in Colombia, Chile, and Mexico, where it has required lowering the cutoff points according to the level of education (Custodio et al., 2020a).
- Rowland Universal Dementia Assessment (RUDAS): A short cognitive test developed to minimize cultural and educational impact in Australia. It measures executive function, visuospatial, praxis, gnosis, recent memory, and verbal fluency. The educational component does not influence it. RUDAS has shown greater sensitivity and specificity than MMSE to differentiate MCI vs. dementia. However, it requires adjusting its cutoff points in each population where it will be applied (Custodio et al., 2020b; Goudsmit et al., 2018; Mateos et al., 2005; Nielsen et al., 2016).
- Stick design test (SDT) specifically evaluates praxis and visuospatial function. Jazmin de Paula et al. compared the MMSE to evaluate the visuospatial function in Brazil. There were no significant differences between the application of the clock drawing vs. SDT. The advantage described for the stick design test was that it had a minor association with formal education (de Paula et al., 2013).
- Short Portable Mental Status Questionnaire (SPMSQ): This test assesses short and long-term memory, orientation, information about daily events, and calculation. Martinez de la Iglesia et al. (de la Iglesiaa et al., 2001) describe a cutoff point for the SPMSQ-VE of three or more errors for people who know how to read and write and four or more errors for illiterates in a Spanish population. However, in this study, they were not classified and correlated by schooling; the same author recommends applying different cutoff points depending on schooling.
- Vellore Screening Instrument for Dementia—Patient/Informant (VSID-P/VSID-I) is a tool divided into two sections. One assesses the patient and the other the informant on cognitive domains and functionality. It sets memory, aphasia, apraxia, agnosia, and loss of executive function, and the questions focus on life activities. It has no cutoff points and was studied in a population in southern India (Stanley et al., 2009).
- The papadum test evaluates visuospatial function and planning. It includes two versions: paper and actual papadum. In both cases, individuals need to imagine that they have to share the papadum among six members of their family. Then, they show the evaluator how they will tear the papadum so that the six members receive an equal share (Crombie et al., 2023).
- The Dementia Arabic Scale is a newly constructed scale that quantitatively assesses orientation to time, persons, place, memory (including registration and repetition), attention, executive function, speech and language assessment, and judgment. Sometimes, the evaluation includes a differentiative task for literate and illiterate participants. The test also considers the evaluation of social cognition with eight marks in which the informant is asked about the patient’s behavior and perceptual motor impairments (Farghaly et al., 2021).
- Free and selective reminding test—Picture version is an episodic memory test adapted to Latin American populations, and consists of six sequential phases: (1) image identification, (2) interference tasks, (3) free recall, (4) cued recall of images that were not previously recalled, (5) selective reminding of images that were not previously recalled with a category cue, and (6) delayed recall at approximately half an hour (both free and cued recall). Phases 2 through 5 are repeated thrice during learning (Montesinos et al., 2022).
- The Clock Drawing test is a brief tool that demands a range of cognitive domains such as visuospatial abilities, concentration, and auditory comprehension. The task involves asking the participant to draw and copy a clock’s face and then draw the hands to indicate a particular time.
- Brazilian Indigenous Cognitive Assessment (BRICA) is an adaptive version of the Kimberley Indigenous Cognitive Assessment (KICA-Cog). This short version was developed in Australia for indigenous older adults without schooling. They evaluate cognitive domains such as memory, verbal comprehension, visual nomenclature, recall, and praxis. Its cutoff point is 31/32, with high specificity and sensitivity.
- Arabic version of the Test of Nine Images (A-TNI93)

### 3.2. Cognitive Domains Are Measured in the Reported Brief Cognitive Assessments

Table S2 (Supplementary file) summarizes the different cognitive domains most evaluated on the other brief cognitive assessments analyzed in Table S1 (Supplementary file). Eight tests are presented with a lasting less than 10 min: Fototest, SPMSQ (Pfeiffer questionnaire), SDT (stick design test), Mini-Cog Test, M@T (memory attention test), GPCOG-Br (the Brazilian version of the General Practitioner Assessment of Cognition), Papadum test, and CASI-S (The cognitive abilities screening instrument, short version). Between these tests, memory, orientation, visuospatial, and visuo-construction are the predominant cognitive domains evaluated. Language, attention, and executive function are less considerate than the other domains. In addition, only GPCOG-Br has questions designed for relatives, increasing accuracy. The number of items is primarily reduced (average of six items). Again, GPCOG-Br is the test with more items but only assesses three domains. Fototest, another specific cognitive assessment design for illiterate and low-educated people, focuses the evaluation on memory and language. CASI-S evaluated orientation, memory, and language. In contrast, the SDT, M@T, and Papadum test assess only two domains.

On the other hand, nine tests have an evaluation time increase than 10 min: MMSE (Mini-Mental State Examination), RUDAS (Rowland Universal Dementia Assessment Scale), Prueba Cognitiva Leganes (Prueba Cognitiva Leganes), MoCA (Montreal Cognitive Assessment), FCSRT (Free and Cued Selection Reminding Test), KICA-cog (Kimberley Indigenous Cognitive Assessment tool for older indigenous Australians), DAS (Dementia Arabic Scale), VSID (Vellore Screening Instrument for Dementia), and IQCODE (Informant Questionnaire on Cognitive Decline in the Elderly). These tests evaluate more domains such as memory, orientation, language, visuospatial, and executive function—nonetheless, the number of items increases (average of 12). Between the brief cognitive assessments, only VSID, DAS, and IQCODE have specific questions for relatives to increase accuracy with an informant.

Attention is the cognitive domain least evaluated among all the reviewed tests. In contrast, memory, language, and orientation are the most estimated domains. Other tests were not considered because they assess only a single cognitive domain (executive function), such as the INECO frontal screening (Bruno et al., 2015; Torralva et al., 2009). Nonetheless, it shows a good quality compared to RUDAS, clarifying the importance of this cognitive process (Custodio et al., 2020b). Finally, the brief cognitive screening tests with more than one cognitive domain task influenced by educational level were MMSE and MoCA. RUDAS in the domain of visuo-construsction (cube drawing) seems to be affected in populations with low rural education or by socio-cultural aspects.

Table S3 (Supplementary file) shows tasks in every cognitive domain that are not influenced by the level of schooling. For example, the cognitive domains more frequently evaluated were memory and orientation. The PCL, DAS, MMSE, MoCA, CASI-S, KICA-cog, Fototest, FCSRT, M@T, RUDAS, and IQCODE include memory assessment with simple tasks for people with low education; meanwhile, orientation is evaluated in PCL, MMSE, MoCA, KICA-cog, SPMSQ, CASI-S, GPCOG, DAS, M@T and RUDAS. We identified that the digit span, memory, verbal fluency, and cube copy from MoCA may not be influenced by educational level, whereas the MMSE is free of the influence of education on orientation, memory, and visuo-construction tasks. RUDAS has been determined to be free from educational bias in five of six cognitive tasks. (visual spatial orientation, ideomotor praxis, judgment, memory, and verbal fluency).

## 4. Discussion

### 4.1. BCS Tools Useful for Low-Educated and Illiterate Populations from LMIC

Our systematic review shows BCS tools in low-educated and illiterate populations for detecting MCI and dementia with suitable psychometric properties and compared with a gold standard of diagnosis had heterogeneity to setting, diagnosis criteria for dementia, sample size and carried out in Eastern countries, and only six publications involved the LAC population. Of the total number of studies included, only nine were conducted in a community setting. This represents a valuable opportunity to improve future studies in that diagnostic accuracy for BCSs varies in community and clinical settings (Paddick et al., 2017).

Considering the difficulty of assessing cognition in a primary care setting with diverse backgrounds under a standardized protocol (Lorentz et al., 2002; Takada et al., 2006), correct dementia symptom identification and diagnosis is challenging in LAC countries, especially considering BCSs were initially developed for highly educated participants (Julayanont & Ruthirago, 2018) and usually language adapted. Nonetheless, several efforts have been made to establish BCSs for illiterate populations in Peru (Custodio et al., 2020b; Zegarra-Valdivia et al., 2019; Zegarra-Valdivia et al., 2023b) and Brazil (Damasceno et al., 2005; Giacominelli et al., 2017). In contrast, other countries in the region remain behind.

### 4.2. Cognitive Domains Assessed in LMIC’ss BCS Tools

Among the articles reviewed, the predominant domains to assess low-educated and illiterate populations are memory, orientation, visuospatial function, and verbal fluency; domains may require less reliance on advanced reading and writing skills. This distinction is crucial for the cognitive assessment of this population, as it could reduce the rate of false positives and subsequently, the overdiagnosis of dementia. (Ramos-Henderson et al., 2022; Ranson et al., 2019). A significant problem with BCSs that use orientation such as MMSE is associated with the possibility of detecting dementia in early or moderate stages but not MCI (Custodio et al., 2021, 2020b; Kosmidis, 2018); additionally, among MMSE tasks, only 3 of its 30 points assess memory, and 4 of them are dependent on literacy: reading and obeying the command “close your eyes”, writing a sentence, copying the figure of the pentagons, and the calculation task, making it inappropriate for illiterate subjects (Pellicer-Espinosa & Díaz-Orueta, 2022). Important domains are less considered, such as executive function (only six studies exploring it: Fototest, RUDAS, MoCA, KICA-cog, VSID, and IQCODE), attention (SPMSQ and MMSE), and praxis (SDT, RUDAS, KICA-cog, and VSID). It is necessary to point out that evaluating these domains does not require language or reading skills. BCSs that include executive functions have better psychometric properties (Custodio et al., 2020b), particularly in subcortical dementia, such as Parkinson’s disease dementia; dementia with Lewy bodies (Jory et al., 2013); vascular dementia (Custodio et al., 2022b); cognitive impairment associated with multiple sclerosis and cortical dementia, for example, behavioral variant frontotemporal dementia (bvFTD) (Custodio et al., 2016; Gleichgerrcht et al., 2011); and language variant primary progressive aphasia (Jory et al., 2013). Some studies suggest an early decline in conflictive instructions and motor inhibitory control in patients with prodromal AD (Kim et al., 2010; Moreira et al., 2014), and recently, other studies suggest that executive function deficits are a crucial feature of the MCI phenotype linked to the risk of progression to dementia in Alzheimer’s clinical syndrome, predicting 63% of the variance of the conversion (Junquera et al., 2020; Parra et al., 2022). These findings are consistent with those supporting that category generation should be included in cognitive composites to predict cognitive impairment (Giacominelli et al., 2017; Peter et al., 2016; Zegarra-Valdivia et al., 2022). However, a failure in the verbal fluency category may indicate language impairment (Alegret et al., 2018). This necessitates that the assessment to be implemented should encompass an appraisal of the broadest possible range of cognitive domains, and if feasible, it should be contrasted with the individual’s demonstrated functionality. Additionally, it is conceivable that other pertinent processes are not being considered, such as those involving social cognition (Zegarra-Valdivia et al., 2023c), for which, along with the aforementioned cognitive processes, their deficits could have a significant impact on individuals’ daily lives. Briefly, the tasks involving reading, writing, calculating, drawing, praxis, visuospatial, and visuo-constructive skills imply particular difficulties for low-educated and illiterate individuals, while executive functions (24) like naming, orientation, and memory are domains less affected by educational level (Pellicer-Espinosa & Díaz-Orueta, 2022).

### 4.3. Cognitive Domains and Functionality Assessed in LMIC

Diagnostic accuracy increases if the cognitive component is correlated with functionality performance based on activities of daily living (ADL). Cognitive complaints can not be self-reported by the person; however, a witness or close relative can inform it, so it has been suggested to include the informant as part of the clinical evaluation as Stanley et al. describe in the VSID (Stanley et al., 2009). Another strategy to assess visuospatial function in illiterate people is to create shapes with common elements under simple instructions. SDT could replace the drawing clock, using sticks or phosphors to assemble figures with different orientations. The main advantage of the SDT is that the educational component does not influence it (de Paula et al., 2013). Additionally, since executive functions are essential to maintain independence in daily living tasks, this reinforces the idea that impairment in executive function tasks is directly related to a loss of autonomy in instrumental ADL (Junquera et al., 2020). So, the correlation between cognitive performance and functionality increases diagnosis accuracy. As with the studies analyzed, it is recommended that the tests be adjusted to the characteristics of the population.

### 4.4. Cognitive Domains Assessed in LAC’s BCS Tools for Dementia’s Specific Types

Age, education, gender, sociodemographic, or cultural biases must be reduced by considering appropriate adaptations or specific task designs in BCS tools. These approaches may increase the sensibility and specificity of cognitive and dementia screening tools. Among the articles reviewed, the RUDAS shows acceptable indicators for low-educated and illiterate populations and has good diagnostic performance for detecting dementia in different socio-cultural settings. Compared to other brief screening instruments, the advantages of the RUDAS include its limited bias in people with little or no formal education and a minimal need for cultural or language adaptation (Nielsen & Jorgensen, 2020).

Depending on the type of dementia, clinical manifestations would vary; symptoms and signs may not be included in a single screening battery, highlighting the importance of taking a complete medical history with the accompaniment of a reliable informant (Parra et al., 2023). Defining a single neuropsychological test as optimal for a population with low schooling is hard (Van Den Dungen et al., 2012). We underscore the importance of utilizing a comprehensive suite of tools that evaluate cognitive processes, executive functions, social cognition, and overall functionality, with benchmarks tailored to the local context for a more accurate assessment of neurocognitive health (World Health Organization (WHO), 2019). Finding a single instrument that encompasses all these aspects or even a concise test that can assess various processes effectively is challenging (Mitchell et al., 2011).

We recommend prioritizing evaluating functionality and general cognition as primary indicators in the initial assessment phase. Subsequently, complementary evaluations should focus on measuring higher-order processes such as executive functions and social cognition to provide a holistic view of an individual’s cognitive health. In this sense, the M@T showed adequate psychometric properties to discriminate between controls, patients with MCI, and patients with early AD in a sample of individuals with low levels of education. Also, they found that the performance of M@T is higher than MMSE and CDT for discriminating both AD vs. amnestic mild cognitive impairment (aMCI) and aMCI vs. controls (Custodio et al., 2017). Recently, the M@T was strongly correlated with typical changes in brain magnetic resonance imaging (Custodio et al., 2022a) and cerebral spinal fluid biomarkers and had good discrimination of neurodegeneration. In LMICs, the M@T may be a cost-effective screening tool for aMCI and dementia caused by AD (Custodio et al., 2023).

Finally, the clinicians need to evaluate the BCS tools, determine the scope and limitations of the cognitive domains analyzed, and interpret with caution when used in low-educated and illiterate older adults. This systematic review recommends specific BCS tools free of educational bias’ influence with global cognitive efficiency, such us RUDAS, GPCOG, and VSID, and BCS tools suggesting aMCI or early AD, such us M@T. However, this BCS tool would fail to detect cases in which the initial symptom does not include memory loss, such as vascular dementia or bvFTD.

### 4.5. Limitations

This research is not exempt from limitations; for example, the initial search and subsequent election of manuscripts were focused on the English and Spanish languages. It is essential to know that different approaches in non-English language are developing in populations with high illiterate populations like India or China. Another limitation is that the number of participants in the studies reviewed recruited is relatively tiny, and age differences between control and clinic groups were identified. On the other hand, do not forget that cultural elements between countries and continents remain essential in these studies. Finally, most studies did not report all the psychometric properties of the assessed screening tools, and this systematic review did not include an analysis of functionality based on ADL. Since executive functions are essential to maintain independence in daily living tasks, this reinforces the idea that impairment in executive function tasks is directly related to a loss of autonomy in instrumental ADL (Junquera et al., 2020).

## 5. Conclusions

In LMICs, the validation of BCS tools for illiterate and low-educated populations remains limited. This study identified thirteen cognitive tests suitable for these populations, emphasizing the significant impact of educational attainment on cognitive abilities. This underscores the critical need for culturally adapted and validated tools that mitigate educational bias.

While these tools provide valuable insights into cognitive decline in low-educated individuals, incorporating functionality assessment into the diagnostic process is essential. Linking cognitive performance with daily living activities significantly enhances diagnostic accuracy, reducing both false positives and false negatives.

There is an urgent need for international consensus to define the most appropriate tools and evaluation protocols, accounting for the cultural, linguistic, and socioeconomic diversity of different regions. Developing representative, country-specific analyses will be pivotal in harmonizing common assessment protocols and facilitating the effective and culturally relevant application of these tools.

We recommend prioritizing functionality and general cognitive assessment as primary indicators during the initial diagnostic phase. These should be complemented by the analyses of higher-order processes, such as executive functions and social cognition, to provide a comprehensive understanding of neurocognitive health. Tools like RUDAS, GPCOG, and VSID have shown effectiveness in these populations, while M@T stands out for the early detection of amnestic MCI and Alzheimer’s dementia. However, further exploration is needed to identify the tools capable of detecting other forms of dementia.

Finally, the development of culturally adapted and validated BCS tools, alongside the implementation of harmonized protocols, represents a critical step toward ensuring equitable and accurate diagnostic evaluation for vulnerable populations in LMICs.

## Figures and Tables

**Table 1:** Search terms.

KEY CONCEPT	SEARCH TERMS USED
Cognitive decline	“Cognitive impairment” OR “cognitive decline” OR “memory impairment” OR “cognitive aging”
Screening test	“Questionnaires” OR “brief cognitive screening” OR “screening test, cognitive battery” OR “neuropsychological battery” OR “Cognitive Assessment” OR “Screening Instrument” OR “Mental Status Tests” OR “Neurocognitive Tests” OR “cognitive test” OR “assess*” OR “screening”
Low literacy	“Low education” OR “education levels” OR “illiterate”, “low-educated” OR “older adults” OR “educational status” OR “educational attainment level” OR “educational background” OR “illiteracy” OR “illiterate*” OR “low literacy”.
Validity	“Validity” OR “test validity” OR “valid”

Note: lists the key concepts and search terms employed in PubMed, Scopus, and Web of Science (September 2023).

## Data Availability

All data are fully available without restriction from the corresponding author upon reasonable request. No original data were generated from this systematic review. All the extracted data are available within the existing tables and references.

## Supplementary Materials

The following supporting information can be downloaded at: https://www.mdpi.com/article/10.3390/bs15020207/s1, Table S1. Brief cognitive test useful for low-educated and illiterate populations. Table S2. Characteristics of brief cognitive assessments for low and illiterate populations. Table S3. Tasks from cognitive domains with minimal educational influence.
