# Supplementary material for: Dementia and Mild Cognitive Impairment Identification in Illiterate and Low-Educated People: Systematic Review About the Use of Brief Cognitive Screening Tools
Source: Behav Sci (Basel). 2025 Feb 13;15(2):207. doi: 10.3390/bs15020207 (PMC11851668; doi:10.3390/bs15020207)
Supplement: Supplementary file 1 [file behavsci-15-00207-s001.zip › behavsci-3286259-supplementary.pdf]

Table S1. Brief cognitive test useful for low-educated and illiterate populations

Author (year)	Tool	Time	Country	Setting	Diagnosis Criteria	Sample size	Mean age	% Female	Education	Diagnostic Accuracy	QA
Martínez de la Iglesia et al. (2001)	Short Portable Mental Status Questionnaire (SPMSQ)	5 min	Spain	Community	ICD10	255	74.5	66.70%	65,5% low education: Illiterate (33,3%), Read and write (32,2%)<br><br>34,5% schooling ≥ 6 years	Cut-off score: ≥ 3 (AUC: 0.891, Se: 85.7; Sp: 79.3)<br><br>According to the level of education:<br>*Illiterate:* Se: 87.5; Sp: 58,3.<br>*Read/write or schooling:* Se: 80,0; Sp: 87,8	LR
García de Yébenes et al. (2003)	Prueba Cognitiva Leganes (PCL)	11 min	Spain	Community	AACD, IPA-WHO criteria; DSM-IV	375	78.6	49.1	Illiterate: 9.1%. Without formal studies 25.6%; < primary school 37.9%; Primary school and more: 27.5%	*Dementia:* AUC: 0.99<br>Cutoff point: ≤ 22 (Se: 93.9%, Sp: 94.7%; diagnostic accuracy: 94.7%).<br>AACD-dementia AUC: 0.904; Cutoff point of ≤ 26 Se: 80.0%; Sp: 84.3%; Diagnostic accuracy: 83.5%.	LR
Carnero-Pardo & Montoro-Rios (2004)	Fototest	<3 min	Spain	Outpatient neurological clinic	DSM-IV-TR	60 (30 Dem; 30 HC)	73.89	60%	Dementia/ HC: Without education: 4/5; <10 years: 20/13.<br>>10 years: 6/12	Cut-off point: < 27 (AUC: 0.94; Se:0.93, Sp: 0.80)	LR
Damasceno et al. (2005)	The cognitive abilities screening instrument, short version: CASI-S	<10 min	Brazil	Neuropsychology Unit; community	DSM-IV; NINCDS-ADRDA	172	59	44.77%	5 years	Cut off point: 24/23 (AUC: 0.87; Se:76.7%, Sp: 86.5%)	LR
LoGiudice et al (2006)	KICA-cog	<15 min	Australia	Community	DSM IV	70: 32 HC; 27 Dem; 11 CIND	72: HC:73.6; DM: 70,7; CIND: 70,9	57.10%	No schooling: HC: 56,3% Dementia: 70,4% CIND: 54,5%	*HC vs. DM:*<br>Cut-off score 31/32 (AUC 0.95; Se: 90,6% Sp: 92,6%)	LR
Stanley et al. (2009)	Vellore Screening Instrument for Dementia–Patient (VSID-P) and for Informant (VSID-I)	VSID-P: <10 min. VSID-I <10 min	India	Outpatient clinics of Geriatric, Neurology, and Psychiatry; community	Clinical Dementia Rating Scale; DSM-IV	191	Hospital Sample: 71,53; Community: 72,5	48.60%	Illiteracy: Hospital sample 37,8%,<br><br>Community sample 53,5%	**VSID-P:**<br>*Hospital sample*: ≤ 9 (AUC 0,91; Se 94.4%, Sp 86.1%).<br>*Community Sample*: ≤11 (AUC 0,81; Se 66,7%, Sp:77,6%)<br>**VSID-I:**<br>*Hospital sample*: ≤ 6 (AUC 0.89; Se 88.9%, Sp 75%)	LR

										Community sample: ≤8 (AUC 0.90; Se: 100%, Sp: 79.2%)<br>**VSID P+I**<br>*Hospital Sample:* Se 83.9, Sp: 96.5%; *Community*: Se 66.7%, Sp: 95.3%.	
Basic et al. (2009)	Rowland Universal Dementia Assessment Scale (RUDAS)	10 min.	Australia	Memory clinic, outpatient geriatric clinic	DSM-IV	151 (60 HC; 33 CIND; 58 Dem)	77.1	68.6%	Literate: 96 %<br>*Without education*<br>HC: 3.3%.<br>CIND: 9.7%.<br>Dementia: 1.9%.<br>*Primary education*<br>HC: 28.3%.<br>CIND: 25.8%.<br>Dementia: 61.1%.	Cut-off score <23/30 (AUC 0.94; Se:87.7, Sp: 90.0)	LR
Youn et al (2011)	Mini-mental state examination (MMSE)	10min	Korea	Community	DSM IV; CDR	100	73.2	100%	Illiterate 50%.<br><br>Literate:<br><br>HC 8,0 years.<br>AD: 7,5 years.	Cut-off score: 24/25 (Se:100; Sp: 4 in illiterates; Se 96% Sp 92% in literates)<br><br>The higher false positive rate (49%) in illiterate elders than in literate elders indicated that the screening cutoff score (24/25) was too high for the illiterate participants.	LR
Lu et al. (2011)	Montreal Cognitive Assessment (MoCA)	10-12min	China	Community	DSM IV; CDR; NINCDS-ADRDA; NINDS-AIREN	8411: 6283 HC; 1687 MCI; 441 Dem	73.0	53.7	5,8 years	*Individuals with no formal education:* 13/14 (Se 80.9% Sp 83.2)<br>*1-6 ye:* 19/20 (Se 83.8 Sp 82.5)<br>*7 or more ye:* 27/25 (Se 89.9%; Sp 81.5%)<br><br>**Applying the adjusted cutoff points**: Se 83.8 % for all cognitive impairments,<br>HC: 82.5%<br>MCI: Se 80.5%<br>Dementia: 96.9%<br><br>*MCI vs HC:* MoCA AUC 0,899 - MMSE 0,842<br>*Dementia vs HC:* MoCA 0,986 - MMSE 0,985.	LR

LoGiudice et al (2011)	KICA-Screen	<15 min	Australia	Community	DSM-IV/ ICD 10	418: 363 Kimberley; 55 North Queensland	60,6 Kimberley 69,6 North Queensland	Kimberley: 55% North Queensland: 63,6%	Received form schooling: Kimberley 60%. North Queensland: 89%	*HC vs Dementia:* **Kimberly**: Cut-point score 21/22 (AUC 0.95; Se 95.6%, Sp 88.6%) **NQ:** Cut-point score 21/22 (AUC 0.87; Se 82.4%, Sp 88.5%)<br><br>Questions that assessed recall, registration, and free recall were highly discriminatory items that correctly classified ***96.7%*** of participants with or without dementia.	LR
Carnero-Pardo et al. (2011)	Fototest	<3min	Spain	Outpatient neurological clinic	DSM-IV	589 (361 NoCi; 106 CInoDEM; 122 DEM)	72.69	56.5	None/incomplete 49,9%; Primary 32,6. Secondary or higher 17.5%.	Dementia<br>Fototest AUC 0,94 <26/27 Se 0,88 Sp 0,87; Eurotest 0,95; VFT 0,90<br><br>CInoDEM<br>AUC Fototest 0,86 <28/29 Se 0,71 Sp 0,84; Eurotest 0,84; VFT 0,78	LR
De Paula et al (2013)	Stick Design Test (SDT)	< 5 min	Brazil	Outpatient public health unit	DSM-IV, NINDS AIREN, CDR	128: 62 HC, 93 mild AD	HC: 75; Mild AD: 75	No specified, but mention no significant differences	HC: 4 years. Mild AD: 4 years.	AUC: 0,756.<br>CDT AUC 0,840.<br>MMSE + SDT= MMSE + CDT 84 % overall prediction (only MMSE 82%)<br><br>Greater accuracy when the clock drawing test was used by itself.	LR
Nielsen et al. (2015)	RUDAS+ IQCODE	10 min + <10 min	Lebanon	Community and nursing homes	DSM-IV / NIA-AA	225	Dementia 81.9 / HC: 77.0	64.00%	Dementia vs. HC:<br>No formal education 61.1%/45.2% %.<br>Primary 17.8% /17.0%%;<br>Intermediate: 12,2%/14,0;<br>Secondary 7.8%/ 13.3%;<br>University 1.1%/ 10.4%	RUDAS AUC: 0.923 Se 0,92 Sp. 0,84; IQCODE AUC: 0,97 Se 0,92 Sp 0,96; OR AUC 0,90 Se 0,96 Sp 0,84; AND AUC 0,86 Se 0,76 Sp 0,97; Weighted sum AUC 0,97 Se 0,86 Sp 0,97	LR
Mateos-Alvarez et al. (2017)	RUDAS	<10 min	Spain	Outpatient Psychogeriatric clinic	ICD10	97 (demented: 35; no	77.9	76.3%	Illiterate: 15.5%;	RUDAS AUC: 0.90<br>Cut-off: 21/22, Se: 94.3, Sp: 72.6; MMSE ACU: 0,889	LR

						demented: 62)			1-5 years: 42.3%; 6-8 years: 27,8 %; 9-12 years: 12.4%; ≥ 13 years: 2.1%	Cutoff: 16/17, Se: 85.7; Sp: 77,4.	
Custodio et al. (2017)	Memory Alteration Test (M@T)	5 -10 min	Peru	Elderly care home centers	DSM-IV; CDR	247	72.69	65.44	low educational level (<4 years of completed formal education)	Early AD vs. aMCI: Cut point 26<br>Se 100.00 % Sp: 97,53%; accuracy: 98.41%.<br>aMCI vs HC: cut point 35, Se 99.17% Sp 91.11%), accuracy 96.99%	LR
Yokomizo et al. (2018)	The Brazilian version of the General Practitioner Assessment of Cognition (GPCOG-Br)	4-6 min	Brazil	Primary care units	DSM-IV	93	Cases: 76,8; HC: 72,5	81%	No formal education: 25,8%; 1-4 years: 45,16%; 5-8 years: 16,13%; >8 years: 12,90%	< 6/7 GPCOG-Br<br>AUC 0.90, Se: 86 Sp: 80	LR
Goudsmit et al (2018)	RUDAS	<10 min	The Netherlands	Geriatric outpatient clinic	NIA-AA; DSM IV TR criteria	144: 42 HC; 44 MCI; 58 dementia	HC: 75; MCI: 75; Dem: 76	HC: 60%; MCI: 73%; Dem: 48%	HC: 1 year; MCI: 0 year; Dementia: 1 year.	HC vs MCI + Dementia:<br>RUDAS AUC 0.81; MMSE AUC 0.77;<br><br>HC vs Dementia:<br>RUDAS AUC 0.89; MMSE AUC 0.85;<br><br>HC+MCI vs Dementia:<br>RUDAS: 0.82; MMSE 0.77.<br>RUDAS Youden's Index <22/21 Se 74% Sp 74 %;<br>MMSE <24 Se 94%; Sp 17%<br><br>MMSE High risk of a false positive diagnosis	LR
Custodio et al. (2020)	Peruvian version of the Rowland Universal Dementia Assessment Scale (RUDAS-PE)	10 min	Peru	Community	DSM-5; CDR	187	70.14	56.15%	Illiterates and without prior literacy experience	HC vs MCI:<br>RUDAS <23, (AUC 0.98 Se 89.06%, Sp :93,33, YI: 0.82%)<br><br>MCI vs D:<br>RUDAS < 19 (AUC: 0.98; Se:95%, Sp:96.83%, YI: 0.92)	LR

Custodio et al (2021)	RUDAS-PE	10 min	Perú	Community	Expert neurological examination and CDR.<br>Controls: CDR 0<br>MCI: CDR 0.5<br>Dementia: CDR 1-2	129:<br>36 HC; 40 MCI; 53 Dem	Site 1: HC: 69,2; MCI: 67,6; Dem: 71,9;<br>Site 2: HC: 71,3; MCI: 67,6; Dem: 73,8	Site 1: MCI, 52.4% Dementia. 54.5%<br>Site 2: MCI, 55.6% Dementia. 54.5%	Illiterate	**Site 1**<br>*MCI vs. HC*<br>AUC 0,82; <22 Se 85,0 Sp 68,2 FPR 24,0; Likelihood ratio + 2.67<br><br>*MCI vs. Dem*<br>AUC 0,99 < 18 S 100% Sp 90,9% FPR 3,0%; Likelihood ratio + 11.00<br><br>**Site 2**<br>*MCI vs. HC*<br>AUC 0,75; <21 Se 81,3 Sp 50,0 FPR 35,0; Likelihood ratio + 1.63<br><br>*MCI vs. Dem*<br>AUC 0,99 < 17 Se 100; Sp 75,0; FPR 10; Likelihood ratio + 4.00	LR
Carnero-Pardo et al. (2018)	Mini-Cog test	3 min	Spain	Community	NIA-AA criteria for MCI<br><br>NIA-AA criteria for Dementia	581:<br>172 HC;94 SCC; 315 CI	63.8;<br>HC: 47.9;<br>SCC: 63.7;<br>CI: 72.5	55.1%	Illiterate: 4.4%<br>< Primary education: 27.7%<br>≥ Primary education: 72.3%	All sample: AUC 0.88 ± 0.01; cut-off point (2/3)<br>Se 0.90 (0.87−0.93)<br>Sp 0.71 (0.65−0.76)<br>+LR: 3.09<br>< Primary education: AUC 0.74 ± 0.05<br>< Primary education: AUC 0.90 ± 0.01	LR
Farghaly et al. (2021)	Dementia Arabic Scale (DAS)	Patients (15-20 min)<br><br>Controls (10-12 min)	Egypt	Clinic	DSM-V	240:<br>120 HC;<br>120 Dem	HC: 66.43;<br>Dem: 68.45	HC: 42,5%;<br>Dem: 39.17%	HC<br>Illiterate: 65%<br>Basic/secondary education: 19.2%<br>Higher Education: 15.8%<br>Dem:<br>Illiterate 65%)<br>Basic/secondary education (24.2%)<br>Higher Education (10.8%)	AUC: 0.964<br>Cutoff ≤95 for literate<br>Se: 100% Sp: 84%<br>Accuracy: 92% PPV 86%<br>cutoff ≤68 for illiterate<br>Se: 87% Sp: 96%<br>Accuracy: 91.5% PPV 95.5%	LR

Montesinos, et al. (2022)	Free and Cued Selective Reminding Test- Picture Version ((FCSRT-Picture)	15 min	Peru	Clinic	NIA-AA criteria for MCI<br><br>NIA-AA criteria for Dementia	187:<br>67 HC;<br>60 aMCI;<br>63 ADD	70.2	56.2%	Illiterate	For the free recall section:<br>AUC: 1.00<br>Cutoff: 16 (to differentiate aMCI and HC)<br>AUC: 0.99<br>Cutoff: 10 (to differentiate ADD and HC, and ADD vs aMCI)<br>Total recall section:<br>AUC: 1.00<br>Cutoff: 26 (to differentiate aMCI and HC)<br>AUC: 1.00<br>Cutoff: 20 (to differentiate ADD and HC)<br>AUC: 1.00<br>Cutoff: 19 (to differentiate ADD and aMCI)	LR
Crombie, Mairi et al. (2023)	Papadum test	<10 min	India	Clinical	CDR	148:<br>HC: 89:<br>Dem: 59	HC: 58.97;<br>Dem: 69.69	HC:56.18 %<br>Dem: 38.98%	HC: 9.58(SD 5.16)<br>Dem: 13.68 (SD 3.62)	Actual Papadum:<br>AUC: 0.778<br>Cutoff: 13.5<br>Se:0.847 Sp: 0.596<br>Youden Index: 0.443<br>Paper Padadum:<br>AUC: 0.782<br>Cutoff: 15.5<br>Se:0.881 Sp: 0.562<br>Youden Index: 0.443	LR
Goudsmit, Miriam, et al. (2020)	Informant Questionnaire for Cognitive Decline in the Elderly (IQCODE)<br>Rowland Universal Dementia<br><br>(RUDAS)	<10 min	Turkey<br>Morocco	Geriatric outpatient clinic and community	NIA-AA;<br>DSM IV TR criteria	129:<br>20 HC;<br>27 intact cognition; 33 MCI; 49 Dem<br>.	HC: 68;<br>Intact cognition: 76;<br>MCI:77;<br>Dem: 78	HC: 60%;<br>Intact cognition: 70%;<br>MCI 76%<br>Dementia 49%	HC: Illiteracy 40%, no education 55%, 1-6 ye 35%, secondary education 10%, tertiary education 0%.<br>Intact cognition: Illiteracy 52%, no education 54%, 1-6 ye 37%, secondary education 7%, tertiary education 0%.<br>MCI: Illiteracy 55%, no education 55%, elementary	Intact cognition vs MCI/dementia<br>AUC IQCODE: 0.86<br>AUC RUDAS: 0.82<br>AUC IQCODE+RUDAS: 0.91<br><br>IQCODE:<br>Cutoff point >3.7<br>Se: 80%<br>Sp: 74%<br>Lr +: 3.10<br><br>RUDAS:<br>Cut off point <21<br>Se: 70%<br>Sp: 78%<br>Lr +: 3.13	LR

									school 33%, secondary education 12%, tertiary education 0%. Dementia: Illiteracy 55%, no education 61%, elementary school 20%, secondary education 12%, tertiary education 6%.		
Kojaie-Bidgoli, (2022)	SPMSQ	10-15 min	Iran	Clinic	DSM-5	156:<br>60 HC;<br>96 CI	74.13 (SD=8.50)	48.1%	4.27 ye (SD:4.70)<br>41 % illiterate	Based on the DSM<br><br>Illiterate: AUC 0.889<br>Cut off point ≥4<br>Se: 0.864 Sp: 88.2<br><br>Literate: AUC 0.948<br>Cut off point ≥3<br>Se: 0.830 Sp: 93.7	LR
Carlos et al. (2023)	Brazilian Indigenous Cognitive Assessment (BRICA)	10-15 min	Brasil	Multiethnic Indigenous community	NIA-AA	141	61.9	58.2%	5.7 (0- 18 years).	CU: ≥ 37 (Se: 58.9%; Sp: 91.2%)<br>CIND: 33.5- 36.5 (Se: 75%; Sp: 64%)<br>D: ≤ 33 (Se: 94.44%; Sp: 99.2%)<br>vs<br>MMSE (Se: 67.9%; Sp: 97.6%)<br>mKICA (Se: 57.1%; Sp: 99%)<br>RUDAS (Se: 60.7%; Sp: 92.3%)<br>It is better to distinguish between CU and D patients.	LR
El- Hayeck et al. (2003)	Arabic version of the Test of Nine Images 93 (A-TNI93)	10-15 min	Lebanese	Community -dwellers	Arabic version of Clinical Dementia Rating (CDR)	265:<br>201 CN<br>40 MCI<br>24 D	-	No dementia: 58.5%<br>Dementia: 50%	Illiterate	Combination of A-TNI93 (FR ≤ 6)<br>Dementia:<br>AUC: 0.93 (Se: 66.7%; Sp: 90.5%)	LR

SPMSQ: Short Portable Mental Status Questionnaire; ICD10: International Classification of Diseases 10th Revision; AUC: Area under curve; Se: Sensitivity; Sp: Specificity; PCL: Prueba Cognitiva Leganes; AACD: Ageing-Associated Cognitive Decline; IPA-WHO: International Psychogeriatric Association-World Health Organization; DSM-IV: Diagnostic and Statistical Manual of Mental Disorders 4ª version; DSM-IV-TR: Diagnostic and Statistical Manual of Mental Disorders 4ª - text revision; HC: Healthy Control; CASI-S: Cognitive Abilities Screening Instrument - Short Form; NINCDS-ADRDA: National Institute of Neurological and Communicative Disorders and Stroke-Alzheimer's Disease and Related Disorders Association; KICA-cog: Kimberley

Indigenous Cognitive Assessment-cognitive assessment section; CIND: Cognitive Impairment No Dementia; VSID-P: Vellore Screening Instrument for Dementia – Patient; VSID-I: Vellore Screening Instrument for Dementia – Informant; RUDAS: Rowland Universal Dementia Assessment Scale; MMSE: Mini Mental State Examination; CDR: Clinical Dementia Rating; MoCA: Montreal Cognitive Assessment; NINDS-AIREN: National Institute of Neurological Disorders and Stroke and the Association Internationale pour la Recherche et l'Enseignement en Neurosciences; NoCi: No Cognitive Impairment; CInoDEM; Cognitive Impairment without Dementia; DEM: Dementia; VFT: Verbal Fluency Test; SDT: Stick Design Test; AD: Alzheimer's disease; CDT: Clock Drawing Test; IQCODE: Informant Questionnaire on Cognitive Decline in the Elderly; BRICA: Brazilian Indigenous Cognitive Assessment; A-TNI93: Arabic version of the Test of Nine Images 93; NIA-AA: National Institute on Aging-Alzheimer's Association; M@T: Memory Alteration Test; GPCOG-Br: Brazilian version of the General Practitioner Assessment of Cognition; MCI: Mild Cognitive Impairment; DSM-5: Diagnostic and Statistical Manual of Mental Disorders 5th version; YI: Youden's Index; FPR: False Positive Rate; DAS: Dementia Arabic Scale. FCSRT: Picture Free and Cued Selective Reminding Test; RUDAS-P: Rowland Universal Dementia Peruvian Version; SPMSQ; Short Portable Mental Status Questionnaire; CDR: Clinical Dementia Rating. +LR: positive likelihood ratio; PPV: true positives; LR: Low risk.

Table S2. Characteristics of brief cognitive assessments for low and illiterate populations

Time	Tools (n=13)	Subtest/ Items	Orientation	Attention	Memory	Language	Executive Function	Visuospatial & Visuoconstruction	Praxis	Informant questionnaire?	Functionality	Total score	Schooling dependent items
<10 min	Fototest	3			x	x	x					30	None
	SPMSQ	10	x	x	x							10	None
	SDT							x	x			24	None
	Mini-Cog Test	3		x	x			x				10	None
	M@T	5	x		x							50	None
	GPCOG-Br	15 (4CT)	x		x			x		Yes	x	15	None
	Papadum test*	1					x	x				18	None
	CASI-S	4	x		x	x						33	None
>10 min	MMSE	6	x	x	x	x		x				30	Attention, writing, and reading
	RUDAS	6	x		x	x	x	x	x			30	visuo-construction
	PCL	32	x		x							32	None
	MoCA	8	x		x	x	x	x				30	Visuospatial/ Executive Functions, naming, and attention (subtraction)
	FCSRT	16		x	x							48	None
	KICA-cog	11	x		x	x	x	x	x			39	None
	DAS	12	x	x	x	x	x	x		Yes	x	120105* *	Visuospatial/ Executive Functions
	VSID-P/I	10+10			x	x	x		x	Yes	x	20	None
	IQCODE	26	x		x	x	x			Yes	x	130	None
	BRICA	11/16	x		x	x	x		x			39	None
	A-TNI93	9			x							27	None

SPMSQ: Short Portable Mental Status Questionnaire; SDT: Stick Design Test; M@T: Memory Alteration Test; GPCOG-Br: Brazilian version of the General Practitioner Assessment of Cognition; C-AD8: Questionary to the informer AD8; CASI-S: Cognitive Abilities Screening Instrument - Short Form; MMSE: Mini-Mental State Examination; RUDAS: Rowland Universal Dementia Assessment Scale; PCL: Prueba Cognitiva Leganes; MoCA: Montreal Cognitive Assessment; FCSRT: Free and Cued Selective Reminding Test; KICA-cog: Kimberley Indigenous Cognitive Assessment-cognitive assessment section; DAS: Dementia Arabic Scale; VSID-P: Vellore Screening Instrument for Dementia – Patient; VSID-I: Vellore Screening Instrument for Dementia –

Informant; IDEA: Identification and Intervention for Dementia in Elderly Africans; IADL: IDEA-instrumental activities of daily living; IQCODE: Informant Questionnaire on Cognitive Decline in the Elderly; BRICA: Brazilian Indigenous Cognitive Assessment; A-TNI93: Arabic version of the Test of Nine Images 93. *: Also known as Pizza test depending on the cultural context in which it is used; **: First score for literate, second score for illiterate.

Table S3. Tasks from cognitive domains with minimal educational influence.

Domain	Task	Test
Orientation (different tasks)	What date is today?<br>What day of the week is today?<br>What time is it?<br>What town are we in?<br>What month of the year are we?<br>Are we in a period of high or low water in the rivers in this region?<br>What is this place where we are?<br>What is your home address?<br>How old are you?<br>Full date of birth<br>Mother's name<br><br>Where are we now?<br>What is your phone number?<br>What is your address? - only if you don't have a phone<br>Who is the current president of the government?<br>Who was the previous president of the government?<br>What are your mother's two surnames?	PCL<br>MMSE<br>MoCA<br>KICA-cog<br>BRICA<br>BRICA<br>BRICA<br>SPMSQ<br>CASI-S<br>GPCOG-Br<br>DAS<br><br>*M@T includes temporal orientation<br>*RUDAS includes visuospatial orientation
Attention	Digit Span (5 forward, three backward)<br><br>Subtraction by three starting at 20	MoCA<br>FCSRT<br>DAS<br>SPMSQ
Memory	Naming, immediate free recall, delayed recall, and logical memory	PCL<br>DAS<br>BRICA
	Registration/encoding and delayed memory recall	MMSE<br>MoCA<br>CASI-S<br>DAS
	Recognition and naming, registration, free recall, and cued recall	KICA-cog<br>Fototest<br>FCSRT

		BRICA<br>A-TNI93
	Episodic (encoding, free recall, and cued recall) and semantic memory	M@T<br>BRICA
	I want you to imagine that we are going shopping. Here is a list of grocery items. I would like you to remember the following items which we need to get from the shop. When we get to the shop in about 5 minutes, I will ask you what we must buy. You must remember the list for me.<br>● Tea<br>● Oil<br>● Eggs<br>● Soap	RUDAS
	1. Remembering things about family and friends, e.g., occupations, birthdays, addresses<br>2. Remembering things that have happened recently<br>3. Recalling conversations a few days later<br>4. Remembering their address and telephone number<br>9. Learning to use a new gadget or machine around the house<br>10. Learning new things in general	IQCODE
Language (verbal fluency)	I would like you to tell me the names of many different animals. We'll see how many different animals you can name in one minute.	MoCA<br>RUDAS<br>KICA-cog
	Please tell me all the names of men/women you can remember.	Fototest
Executive Function / Functionality	8. Knowing how to work familiar machines around the house<br>12. Making decisions on everyday matters<br>13. Handling money for shopping<br>14. Handling financial matters, e.g., the pension, dealing with the bank<br>15. Handling other everyday arithmetic problems, e.g., knowing how much food to buy, knowing how long between visits from family or friends<br>16. Using their intelligence to understand what's going on and to reason things through the Informant version	IQCODE
	1. Does he/she regularly forget events that have happened recently?	VSID-P/I

	2. Does he/she have trouble remembering where he/she has kept her belongings?<br>3. Does he/she regularly have difficulty finding the right words, or does he/she use the wrong words in conversation?<br>4. Does he/she regularly have difficulty understanding what is said to him/her?<br>5. Does he/she regularly have difficulty in dressing appropriately?<br>6. Does he/she urinate in the appropriate place?<br>7. Does he/she have difficulty recognizing familiar faces?<br>8. Does he/she have difficulty recognizing familiar objects like a key, comb, and spoon?<br>--<br>Judgment: "You are standing on the sidewalk of a street that has much traffic. There is no crosswalk or traffic lights. Tell me what you would do to safely cross to the other side of the street."	 <br><br><br><br><br><br><br><br><br><br>RUDAS
Visuospatial & Visuoconstrucción	Ask the patient to copy a pair of intersecting pentagons.<br><br>Cube copy<br><br>Division of an 18 cm diameter paper circle and being asked to imagine it is a pizza. Participants are instructed to divide the papadum/pizza equally among six people, requiring them to fold and divide the paper into six equal pieces.	MMSE<br><br>MoCA<br><br>Papadum test*
Praxis	1. Open this bottle and pour water into this cup<br>2. Show me how to use this comb.<br>--<br>I am going to show you an action/exercise with my hands. I want you to watch me and copy what I do. Copy me when I do this (i.e., demonstrate -put one hand in a fist and the other hand palm down on the table or your knees and then alternate simultaneously.) Now do it with me. I would like you to keep doing this action at this pace until I tell you to stop - approximately 10 seconds or 5 – 6 sequences. (Demonstrate at a moderate walking pace).	KICA-cog<br>BRICA<br><br>RUDAS

SPMSQ: Short Portable Mental Status Questionnaire; SDT: Stick Design Test; M@T: Memory Alteration Test; GPCOG-Br: Brazilian version of the General Practitioner Assessment of Cognition; C-AD8: Questionary to the informer AD8; CASI-S: Cognitive Abilities Screening Instrument - Short Form; MMSE: Mini-Mental State Examination; RUDAS: Rowland Universal Dementia Assessment Scale; PCL: Prueba Cognitiva Leganes; MoCA: Montreal Cognitive Assessment; FCSRT: Free and Cued Selective Reminding Test; KICA-cog: Kimberley Indigenous Cognitive Assessment-cognitive assessment section; DAS: Dementia Arabic Scale; VSID-P: Vellore Screening Instrument for Dementia – Patient; VSID-I: Vellore Screening Instrument for Dementia – Informant; IDEA: Identification and Intervention for Dementia in Elderly Africans; IADL: IDEA-instrumental activities of daily living; IQCODE: Informant Questionnaire on Cognitive Decline in the Elderly; BRICA: Brazilian Indigenous Cognitive Assessment; A-TNI93: Arabic version of the Test of Nine Images 93. *: Also known as the Pizza test, depending on the cultural context in which it is used.
